# Lime-Phosphorus Fertilizer Efficiently Reduces the Cd Content of Rice: Physicochemical Property and Biological Community Structure in Cd-Polluted Paddy Soil

**DOI:** 10.3389/fmicb.2021.749946

**Published:** 2021-11-19

**Authors:** Xiaolin Kuang, Kangying Si, Huijuan Song, Liang Peng, Anwei Chen

**Affiliations:** ^1^Department of Environmental Science and Engineering, Hunan Agricultural University, Changsha, China; ^2^Hunan Engineering and Technology Research Center for Irrigation Water Purification, Changsha, China

**Keywords:** amendment, biodiversity, heavy metals, rice, soil

## Abstract

Due to the biomagnifying effect in the food chains, heavy metals will cause serious harm to the food produced in paddy soil, and then threaten human health. The remediation of soil heavy metals by the addition of amendments is a common method. However, the combination of the two amendments has been less studied and its effect is unknown. In this study, we investigated the effects of different concentrations of a lime and calcium-magnesium phosphate (CMP) amendments metal availability and paddy soil bacteria biodiversity. The experiment proves that the addition of 0.5 and 1.0‰ amendment can effectively reduce cadmium (Cd) availability and the cadmium content in rice to be below 0.2 mg/kg, meeting the national food safety level. The results demonstrate that increasing pH and phosphorous (P) in soil were two important factors decreasing available cadmium. Furthermore, biodiversity analysis of the treated soil showed that the amendment increased biodiversity. Proteobacteria and Chloroflex were the most abundant bacteria at the phylum level, followed by Acidobacterium and Nitrospirae. The abundance of *Bacterodietes*-*vadinHA17*, *Syntrophaceae*, and *Thiobacillus* increased as phosphorous increased. Cadmium passivation might induce those species.

## Introduction

China’s soil safety faces severe challenges. According to reports, more than 82.8% of the soil has been contaminated by heavy metals, with the highest proportion consisting of cadmium (Cd) (7.0%) (Zhao et al., 2015). Cadmium has a strong ability to migrate. Cadmium entering the soil is easily absorbed by crops and enters the human body through the food chain, endangering human health (Singh et al., 2021). Due to various reasons, in recent decades the soil in southern China’s paddy fields has become increasingly acidified (Shi et al., 2020). Soil acidification leads to cadmium becoming significantly more soluble in the soil, increasing its availability to plants (Zhen et al., 2019). Rice, one of China’s most important food sources, has a strong ability to accumulate cadmium (Mao et al., 2019). Long-term consumption of rice containing high levels of cadmium can harm the human body, for example, by causing “Itai-itai disease” (Li et al., 2019). Consequently, it is imperative to find ways to reduce cadmium concentration in paddy field soil, particularly in southern China.

Currently there are four main strategies for reducing cadmium accumulation in rice: (1) decrease cadmium bioavailability in the soil; (2) reduce cadmium uptake by rice roots; (3) decrease the cadmium transport ratio in rice transport systems; and (4) Screen rice varieties with low cadmium accumulation. The selection of rice varieties is very important; previous studies have found that the response of rice to amendment and Cd uptake depends largely on rice varieties (Saengwilai and Meeinkuirt, 2021; Xu et al., 2021) explored the influence of rice varieties on the cadmium reduction effect of amendment and found that the accumulation of cadmium varies greatly between different rice varieties. Therefore, some efficient technologies have been founded based on these strategies. Treatments with silicone, lime (CaCO_3_), and calcium-magnesium phosphate (CMP) fertilizers can reduce cadmium activity in the soil, thereby preventing cadmium from moving from the soil to the rice (Li et al., 2018; Rehman et al., 2019). In a high pH environment, cadmium mainly exists in the forms of CdOH^+^, Cd_2_(OH)^3+^, and Cd(OH)_2_, which reduces its bioavailability (Peng et al., 2016). The PO_4_^3–^, CO_3_^2–^, S^2–^, and SiO_4_^4–^ anions can induce low-resolution minerals after reacting with Cd^2+^. Other amendment properties such as clay with high adsorption capacity or manure with strong complex ability on the Cd^2+^ were considered as an additional supplement (Sun et al., 2020). Therefore, many normal efficient amendments for cadmium-polluted soil include the alkaline substance (high pH) and some cations (Li et al., 2021; Wang et al., 2021).

The above-mentioned amendments not only reduce the activity of cadmium but also reduce rice’s absorption of elements such as iron (Fe), manganese (Mn), zinc (Zn), calcium (Ca), copper (Cu), and magnesium (Mg). Therefore, the formation of Fe/Mn oxide film in rice root is reduced, thus reducing the resistance to cadmium in rice. However, the transport pathway of cadmium in rice is similar to those of some other nutrients. For example, a range of micronutrient transporters from the family of zinc-regulated transporter/iron-regulated transporter-like proteins (ZIP, such as AtIRT1 and TcZNT1/TcZIP4) and natural resistance-associated macrophage protein (NRAMP, such as Nramp5) can also transport cadmium in rice and *Arabidopsis* (Siqi et al., 2019; Guha et al., 2020). Therefore, cadmium can be controlled through the intake of trace elements (Liu et al., 2018). Yang Y. et al. (2018) reported that low concentrations of lime can increase cadmium in rice while high concentrations of lime decrease cadmium. Regrettably, their research did not clearly explain the relationship between amendment amount, essential elements content, and cadmium concentration.

Although soil amendments are widely applied to decrease cadmium content in rice, their effect on the structure of cadmium-contaminated soil’s biological communities is rarely reported. Levels of soil metals are highly correlated to bacterial species. Sulfur-reducing bacteria, iron/manganese-oxidizing bacteria, organic matter-degradation bacteria, and lactic acid bacteria can greatly influence cadmium content in the soil. Stroud et al. (2014) reported that manganese bioaccessibility positively correlated to *Acidobacterium* abundance, but negatively correlated to *Holophaga* abundance in the acidic sulfate soil. Cadmium was found to be mineralized by alphaproteobacteria and chloroplast classes in rice crust soil, making it biologically unavailable (Song et al., 2019). Paddy soils contaminated with cadmium usually have low pH and lack phosphorus. Therefore, amending soil with lime and CMP can greatly change environmental parameters and alter biological community structure. However, the relationship of biological composition with metal bioaccessibility after treatment with an amendment remains to be investigated further.

Lime and CMP are two typical amendments for cadmium-polluted soil. In our experiment, an amendment consisting of both lime and CMP was applied to investigate the effect of amendment concentration on essential soil elements and paddy roots. Soil biological community structure after treatment with different concentrations of amendments and the relationship between biological species and metal bioavailability were explored.

## Materials and Methods

### Site, Amendment, and Rice Cultivar

The study began in June 2018 in Chang Feng Village, Liu Yang City, Hunan Province, China. The main source of pollution in paddy fields was wastewater from mining areas. The concentration of cadmium in the soil was 0.3 mg kg^–1^, a level considered mildly polluted. The basic physical–chemical properties are shown in Table 1.

**TABLE 1:** Physical-chemical properties of soil in a paddy field.

Total Cd (mg kg^–1^)	Av Cd (mg kg^–1^)	pH	OM (g kg^–1^)	Alkaline-N	Olsen-P(g kg^–1^)	Olsen-K
1.20 ± 0.03	0.21 ± 0.01	6.30 ± 0.08	45.95 ± 2.68	80.50 ± 4.75	22.44 ± 1.49	62.25 ± 3.41

This study used CMP + lime (Fat Baby No.7, FB7) configured in this laboratory as an amendment. Table 2 shows the detailed components of FB7; all of the materials came from Ma Po Mountains, Changsha City.

**TABLE 2:** Material and concentration about amendments.

Marker	Material	Amount
CK	Blank control	
0.2‰	Calcium magnesium phosphate + Lime	315 kg/hm^2^ + 105 kg/hm^2^
0.5‰	Calcium magnesium phosphate + Lime	787.5 kg/hm^2^ + 337.5 kg/hm^2^
1.0‰	Calcium magnesium phosphate + Lime	1,575 kg/hm^2^ + 675 kg/hm^2^

*The column of concentration containing 2 concentration values corresponds to the column of material.*

The rice cultivar was Zhongzao 39, cultivated by the China Rice Research Institute. Its entire growth period was 112 days.

### Experimental Design

The field experiment consisted of four different amounts of FB7 (Table 2). Each concentration treatment was repeated three times in parallel; the area of each treatment was 20 m^2^. Before transplanting rice, FB7 was applied to the soil, mixed thoroughly, and left alone for a week to reach equilibrium. A concrete enclosure was constructed to separate each plot and to avoid mixing irrigation water and fertilizer. The experimental group and control group were randomly distributed, and rice cultivation and management were consistent with local practices for growing rice. The paddy field was flooded and plowed on July 5, 2018, planted on July 12, 2018, and harvested on November 5, 2018.

### Sampling

Five soil samples were randomly selected from each plot for mixing. All experimental samples were air-dried naturally in the laboratory. Biological debris was collected at the ripening stage, and the plant sample in each plot was a mixture of five random samples. First, the rice samples were washed with distilled water. Second, the rice samples were heated at 105°C for 60 min. Finally, the rice samples were dried at 70°C to a constant weight. All samples were ground and sieved before the analysis.

### Sample Analysis

The air-dried soil was sieved (pore dimeter < 0.15 mm) and then the 0.500 g soil was put into a digestion tube. Plant samples were soaked overnight in 4.5 mL of concentrated HCl and 1.5 mL of concentrated HNO_3_, and then digested. This mixed sample was dissolved at 90°C for 1 h and then up to 150°C until the solution inside boiled, after which the sample was removed and cooled down. After the sample was cool, 5 mL of concentrated HClO_4_ was added. It was digested again at 190°C for 2 h and then up to 220°C until about 1 mL of sample was left. The digested sample was then diluted to 25 mL with distilled water and filtrated into a plastic bottle. The concentrations of cadmium in the digested solution were measured with ICP-OES (ICPMA 8300, Perkinelmer).

The total concentration of metal in a plant was measured as follows. The 1.0 g (dry weight) rice and 0.5 g (dry weight) root were digested with 15 mL of concentrated HNO_3_. The cooled digested solution was diluted to 25 mL. The concentration of cadmium, iron, manganese, copper, and zinc in the filtrate was measured using ICP-OES (ICPMA 8300, Perkinelmer).

The pH value, organic matter, cation exchange capacity, phosphorous, Olsen-K, Alkaline-N, and Total-N of the soil were measured according to Yang’s method (Yang et al., 2008). The crystal structures of soil samples were recorded with a diffractometer (TTRIII, Rigaku, Tokyo, Japan), and the surface structure and morphology of the paddy soils were characterized by a scanning electron microscope-energy dispersive spectrometer (SEM-EDS, JEM-1230 HC, JPN).

### Bacterial Richness and Diversity

Soil in the rhizosphere area was collected and mixed from five sites at every plot. Detailed measurements of bacterial species richness and diversity is in Supplementary Material. Sequencing data generated from this study has been deposited in the National Center for Biotechnology Information (NCBI) with the project accession number PRJNA761207.

### Statistical Analyses

Statistical analyses consisted of one-way analysis of variance (ANOVA) using SPSS version 11.5 (SPSS Inc., Chicago, IL). Using the least significant difference (LSD) method, the difference was considered to be statistically significant when *P* < 0.05.

## Results and Discussion

### Effects of the Amendment on Soil pH and Conductivity

**FIGURE 1:**
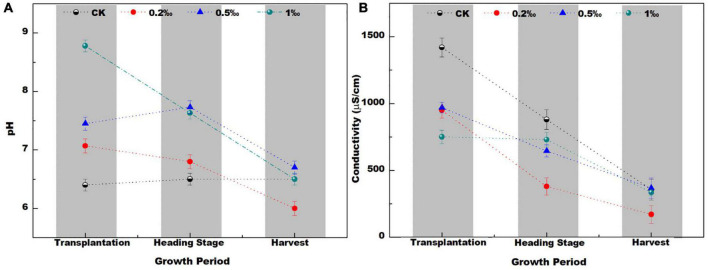
The pH **(A)** conductivity **(B)** in soil with different amendment concentrations at different growth stages of the rice.

The pH in the control check (CK) soil treatment ranged from 6.4 to 6.6 during the three stages (transplantation, heading, and harvest) (Figure 1A). During the transplantation stage, the soil pH value increased from 7 to 9 after the addition of FB7. In the heading and harvest stages, the soil pH was slightly lower than the transplantation stage, but the overall value was still higher than that of the CK treatment. As the amount of FB7 increased, the pH value of paddy soil increased.

The pH value first increased and then decreased with time. This is because lime can increase the pH of the soil (Bouray et al., 2021), but this effect diminishes over time until it returns to its original pH value (Yin et al., 2021). Some groups have also described reacidification of limed soils in the long term (Wang et al., 2009; Rosa et al., 2020). These reports are similar to the results of this experiment—the pH value decreases to a certain extent during the later stage of rice growth. Specifically, the lower pH was found in the treatment with 0.2‰ amendment. In general, the addition of amendment had a great effect on soil pH.

Soil conductivity (EC) decreased sharply as rice growth increased (Figure 1B). Conductivity reflected the ion strength mainly from fertilizer, such as K^+^, NO_3_^–^, and Ca^2+^. The adsorption and sediment of these ions can decrease conductivity (Lin et al., 2005). Notably, conductivity in the treatment with 0.2‰ FB7 was lowest at the harvest stage. It might correspond to the lowest pH and some special soil microbiological communities.

### Effect of FB7 on Cadmium Concentration in the Soil–Root–Rice System

The agronomic information related to rice maturity is shown in Supplementary Table 1. After FB7 was applied, the effective tiller number per plant, biomass, yield, and seed setting rate were significantly increased, but there was no significant effect on 1,000-grain weight. The results showed that amendment could increase the yield and biomass of rice by increasing the effective tiller number (Wang et al., 2020). Moreover, the application of amendment also increased the content of calcium and phosphorus in the soil (Supplementary Table 2). It not only improves soil fertility, but also reduces the concentration of cadmium in rice roots through the competition between Ca^2+^ and Cd^2+^ (Wang et al., 2021).

**FIGURE 2:**
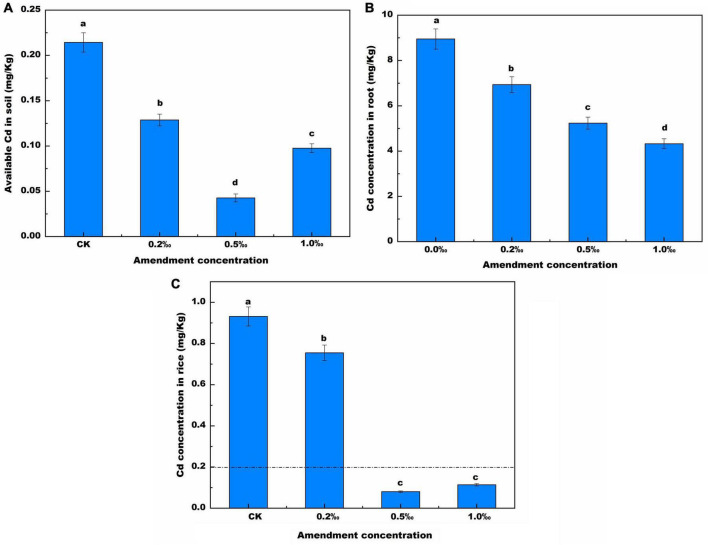
Available Cd in soil **(A)**, Cd concentration **(B)** in root and in the rice **(C)** with different concentration of amendment. Values are means ± SD (*n* = 3). Different letters above the bars indicate the signifcant differences at *p* < 0.05.

The available cadmium content of treated soil was lower than that of untreated soil. (CK) (Figure 2A). The available cadmium concentration was 0.21 mg/kg, and it was only 17.9% of the total cadmium concentration. Compared with the CK soil, the available cadmium concentration decreased by 6.94, 14.33, and 9.74%, after addition of 0.2, 0.5, and 1.0‰ FB7 amendments, respectively. The low available cadmium was attributed to two causes. On the one hand, the solubility of heavy metals decreases as the pH value increases, thereby improving the adsorption capacity of soil particles for heavy metals by increasing the net negative charge of variably charged colloids (Mcbride et al., 2010). On the other hand, the high precipitate effect of CMP for Cd^2+^ results in low available cadmium. The solubility product (*K*sp) for Cd_3_(PO_4_)_2_ was 2.53 × 10^–29^, which is far lower than the *K*sp for Cd(OH)_2_ (6.5 × 10^–14^) and the *K*sp for CdCO_3_ (3 × 10^–12^) (Han et al., 2017). Therefore, the available cadmium concentration increases and then decreases greatly as the FB7 amount increases. The available cadmium concentration was very low, only 0.04 mg/kg, in the treatment with 0.5‰ amendment. Furthermore, similar results were found in the available iron, zinc concentration (Supplementary Table 3). It was attributed to the highest pH value among the treatments being in the soil from the treatment with 0.5‰ FB7 at the harvest stage.

Cadmium content in roots gradually decreased as the amount of FB7 increased (Figure 2B). Cadmium content in roots was 16.68, 11.14, 5.18, and 9.61 mg/kg after addition of 0, 0.2, 0.5, and 1.0‰ FB7, respectively. The total iron, manganese, zinc, copper, and cadmium concentration are revealed in Table 3. The content of zinc and manganese increased, and the content of iron, copper, and cadmium decreased. Compared with CK, after applying the amendment zinc increased by 7.3–31.8 mg/kg while iron decreased by 69.8–86.25 g/kg and copper was reduced by 16.6–42.05 mg/kg.

**TABLE 3:** The concentration of metals in the root of rice.

Treatments	Fe/g kg^–1^	Mn/mg kg^–1^	Zn/mg kg^–1^	Cu/mg kg^–1^	Cd/mg kg^–1^
CK	319.00 ± 8.75 a	151.35 ± 8.66 d	89.90 ± 4.68 b	77.20 ± 3.75 a	8.95 ± 0.35 a
0.2‰	232.75 ± 9.01 d	233.05 ± 7.68 c	121.70 ± 7.55 a	60.55 ± 2.05 b	6.94 ± 0.48 b
0.5‰	249.20 ± 5.94 bc	364.15 ± 5.71 a	97.20 ± 5.65 b	37.16 ± 1.98 c	5.24 ± 0.24 c
1.0‰	244.05 ± 8.35 cd	273.50 ± 7.36 b	99.05 ± 3.21 b	35.15 ± 2.12 c	4.33 ± 0.18 d

*Different lowercase letters indicate significant differences in the content of the same substance between different treatments (P < 0.05).*

Maybe the decrease of metal concentration in the root has relation to the low available metal concentration in soil and the competition effect. Usually, low available metal concentration in the soil induces low concentrations of metals such as iron, copper, and cadmium in the root. Alternatively, the competition effect may be attributed to low cadmium concentration in the root. Cadmium mainly enters the rice root system in the form of ion. Ca^2+^, Mg^2+^, Zn^2+^, and Cd^2+^ are divalent cations with similar physicochemical properties. Therefore, plants often absorb and transport these cations by using the same transport proteins and ion channels, so there is a competition in these ions (Zhang et al., 2019). The large amount of calcium and magnesium in FB7 can occupy the associated ion channels and reduce the root’s cadmium uptake. As shown in Supplementary Figure 1, the application of amendment significantly reduces translocation factor (TF) and bioconcentration factor (BCF).

An interesting trend was found for manganese and zinc, with these nutrients having lower available concentration in the soil and higher concentration in the root after FB7 treatment. Zinc and manganese are common nutrients in rice and their absorption can be enhanced by the application of calcium-containing fertilizers (Luo et al., 2020). The other mechanism might be attributed to the competition effect between zinc and cadmium. Very low available cadmium concentration induces high zinc uptake (Cai et al., 2019). Similarly, manganese and iron demonstrated a competition effect on each other. Therefore, phosphate fertilizer decreased available iron concentration and then increased manganese uptake. However, Mao et al. (2019) reported that phosphate fertilizer would dramatically reduce the availability of manganese and increase the molar ratio of iron to manganese in iron plaques on root surfaces. The difference might be attributed to soil type and the amount of phosphate fertilizer.

The cadmium content in rice was 0.92, 0.76, 0.08, and 0.11 mg/kg (Figure 2C) after addition of 0, 0.2, 0.5, and 1.0‰ FB7, respectively. The cadmium concentration in rice after treatment with 0.5 and 1.0‰ FB7 decreased to less than 0.2 mg/kg, which is the National Food Safety Standard. Specifically, when the FB7 concentration is 0.5‰, the cadmium content in rice is the lowest.

### Effects of Amendment on Soil Microstructure

**FIGURE 3:**
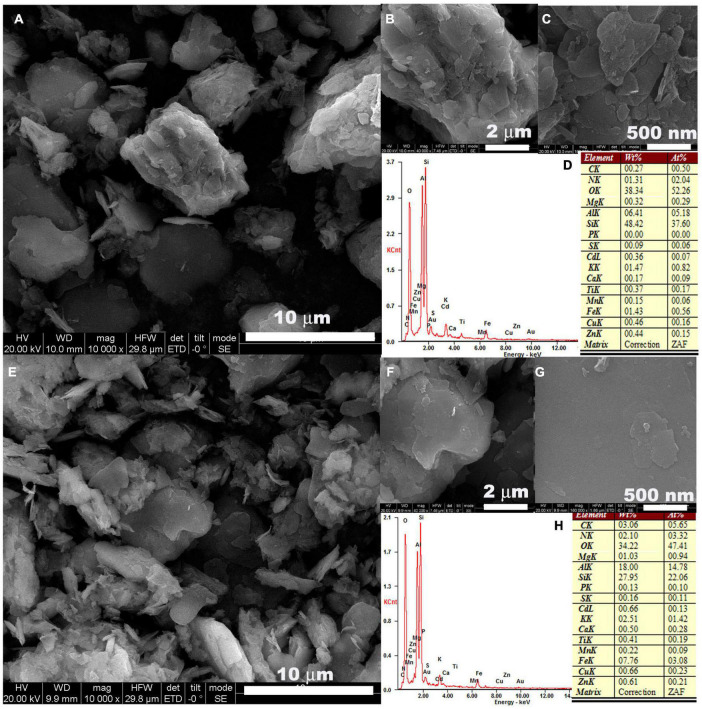
The SEM of soil processed with **(A–C)** 0 and **(E–G)** 1.0‰ amendment, and its corresponding EDS result **(D,H)**.

The typical microstructure of the soil from the CK and 1.0‰ treatment are shown in Figures 3A–H. Soil samples contained a range of minerals, including quartz, muscovite, and kaolinite, which was determined by the X-ray diffraction (XRD) (Supplementary Figure 2). The separated small sheet structure was attributed to the quartz and the aggregated large composites corresponded to the kaolinite and muscovite, as shown in Supplementary Figures 3, 4. There was no obvious variation in the microstructure of soil minerals after treatment with 1.0‰ FB7. However, the surface of the kaolinite became smoother in Figure 3G in comparison with that of Figure 3C. Furthermore, a small amount of phosphorous was found from the surface of the kaolinite in the CK and its concentration increased to 0.13% after processing with 1.0‰ FB7. Some phosphorous was incorporated into the surface of the mineral. The smooth surface might be attributed to the dissolution-precipitation effect of CMP.

### Overview of 16S rRNA Sequencing

In order to study the composition of the microbial community in the paddy soil, we sequenced 16S RNAs. After removing the low-quality readings and trimming the adapters and barcodes, there were 192,952 effective sequences with an average length of 311.85 bp of microbiota generated from the four paddy soil samples: 43,421, 51,084, 49,104, and 49,343 sequences on average for the samples collected from soil treated with 0, 0.2, 0.5, and 1.0‰ FB7 (marked as CK, C2, C5, and C10), respectively. Coverages of 0.966, 0.964, 0.957, and 0.964 were achieved (Table 4). The shape of the rarefaction and Shannon–Wiener curves tended to approach the saturation plateau, showing that the bacterial abundance of this sample was relatively complete (data not shown). The sequences were assigned to 11,160 OTUs of bacteria.

**TABLE 4:** Statistics of the trimmed sequences and alpha-diversity of the bacterial communities.

Sample	Primers	Sequence	Bases (bp)	Mean length (bp)	OUT	Sobs	Shannon	Simpon	Ace	Chao	Coverage
CK	338_806R	43,421	18,103,363	416.990	2,989	3,094	6.858	0.0025	4118.195	4106.610	0.966
0.2‰	338_806R	51,084	21,310,944	417.153	2,943	3,553	6.995	0.0022	4720.416	4691.613	0.964
0.5‰	338_806R	49,104	20,508,293	417.628	2,851	3,747	7.057	0.0023	5157.998	5035.537	0.957
1.0‰	338_806R	49,343	20,636,836	418.207	2,377	3,709	7.044	0.0022	4962.246	4866.319	0.964

Based on the OTUs number, the soil sample from the CK treatment was found to have the richest value, with an OTUs number of 2,989, whereas the samples from 0.2, 0.5, and 1.0‰ FB7 displayed considerably lower richness, with OTUs numbers of 2,943, 2,851, and 2,377. Generally, the Chao1 and Ace indices were used to calculate the OTUs numbers. The experimental results show that the Ace and Chao1 indices of the four samples were negatively correlated with the OTUs numbers. Otherwise, the Shannon indices indicated that the 0.5‰ FB7 treatment showed the greatest diversity (7.057) while the sample of others displayed relatively lower diversity (6.858, 6.995, and 7.044). However, the Simpson index indicated that the CK treatment showed great diversity (0.0025), and the others had relatively lower diversity. Ultimately, the 0.5‰ FB7 treatment was considered to have the highest diversity.

Heavy metals and nutrition are two key parameters affecting biodiversity (Xie et al., 2016). These results indicated that the application of amendment has obvious effects on soil biological community structure. The 0.5‰ FB7 treatment had richer biodiversity than others. This result could be attributed to FB7 optimizing the biological growth environment by increasing fertility and reducing bioavailability of heavy metal, but also destroying its environment to decrease the number of biomes after the addition of too much mineral element.

Karaca et al. (2002) found that when 50 mg/kg of cadmium was added to contaminated soil (sandy loam), the population abundance of bacteria and pseudomonas was significantly increased after also applying sludge and phosphate fertilizer. In addition, their study found that applying red mud, lime, and zeolite to acidic soil contaminated with lead, cadmium, and zinc could effectively improve the number of soil microorganisms. All of these suggest that the addition of amendment leads to an increase in biodiversity.

Gong et al. (2021) found that soil with light heavy metal pollution significantly reduced the diversity of fungi after a large application of amendment. Therefore, excessive amendment would decrease biodiversity. Similarly, the addition of amendment led to the increase and then decrease of biodiversity in this experiment.

**FIGURE 4:**
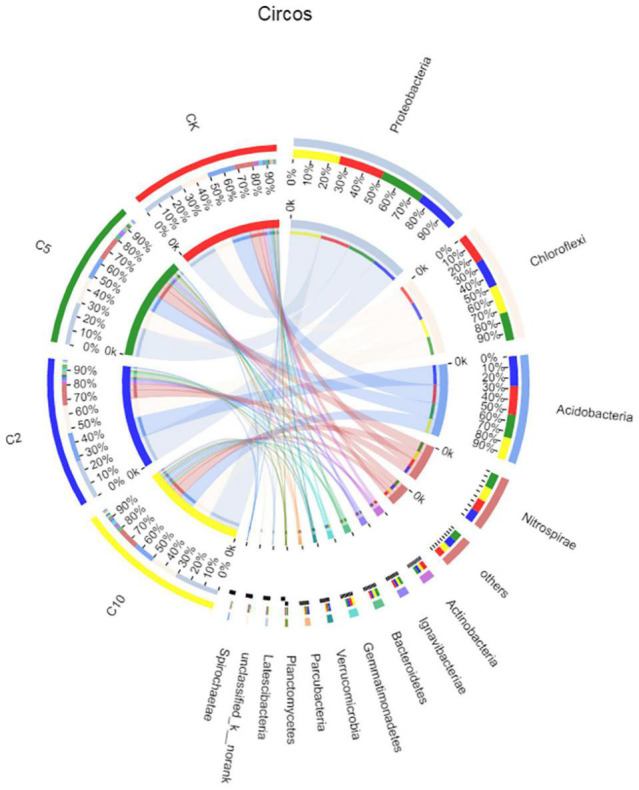
Relationships between samples and phyla are shown in Circos figures. The CK, C2, C5, and C10 is representative of the sample from soil processed with 0, 0.2, 0.5, and 1.0‰ amendment, respectively.

Figure 4 shows the distribution of bacterial phyla in the paddy soil samples. Proteobacteria was the most abundant phylum in all the soil, accounting for 31, 30, 28, and 32% of effective bacterial sequences of the CK, C2, C5, and C10 treatments, respectively. The next most dominant phylum in the CK, C5, and C10 treatments was Chloroflex, with a prevalence of ∼20%. However, in the C2 sample, the second most dominant phylum was Acidobacterium, with a prevalence of 21%. This result might correspond to the strong acidification effect in the soil with 0.2‰ FB7. Consequently, Proteobacteria and Chloroflex were the most abundant phyla, followed by Acidobacterium and Nitrospirae.

Proteobacteria can produce exopolysaccharides, and thus can participate in soil stabilization (Liu et al., 2017). In this study, heavy metals hardly affected the relative abundance of Proteobacteria. The relative abundance of Proteobacteria was only slightly influenced by different levels of heavy metal bioavailability; the main OTUs shared by all samples belonged to the Proteobacteria. Some sequences of Proteobacteria, such as *Halomonas* and *Halovibrio* sp., can also assist carbonate mineral formation (Heijs et al., 2006). Therefore, Proteobacteria have a high detoxifying effect with regard to contamination by heavy metals. Other research has suggested that Proteobacteria offer some resistance to pollution from various heavy metals (Zhu et al., 2013), and this can explain the abundance of this phylum in all samples.

### Correlation Between Bacterial Community, Environmental Parameters, and Phyla

**FIGURE 5:**
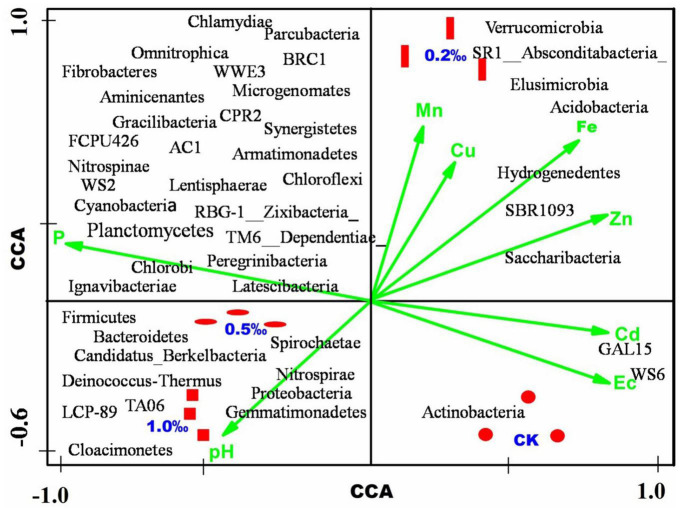
Canonical correspondence analysis (CCA) of the microbial community, environmental parameters, and samples.

In this study, eight physical and chemical indexes (pH, EC, available phosphorus, available iron, manganese, copper, zinc, and cadmium in soil) were analyzed and the relative contribution of the bacterial community at the phylum level was evaluated (Figure 5). The available phosphorous and pH were positive corresponded to the C5 and C10 treatments, which were treated with high concentration FB7. The EC and available cadmium were positively related with the CK treatment, and the available manganese and copper were related to the C2 treatment. The environmental parameters results indicated that the high pH and phosphorous effectively decreased the available metal concentration in the soil, such as manganese, copper, iron, zinc, and cadmium. Environmental parameters regarding phosphorous contributed positively to Peregrinibacteria, RBG-1, Ignavibacteriae, Nitrospinae, Firmicutes, and Latescibacteria. The pH level contributed positively to the Proteobacteria, Cloacimonetes, and Gemmatimonadetes. The available cadmium and EC were positively contributed to the GAL15 and WS6.

**FIGURE 6:**
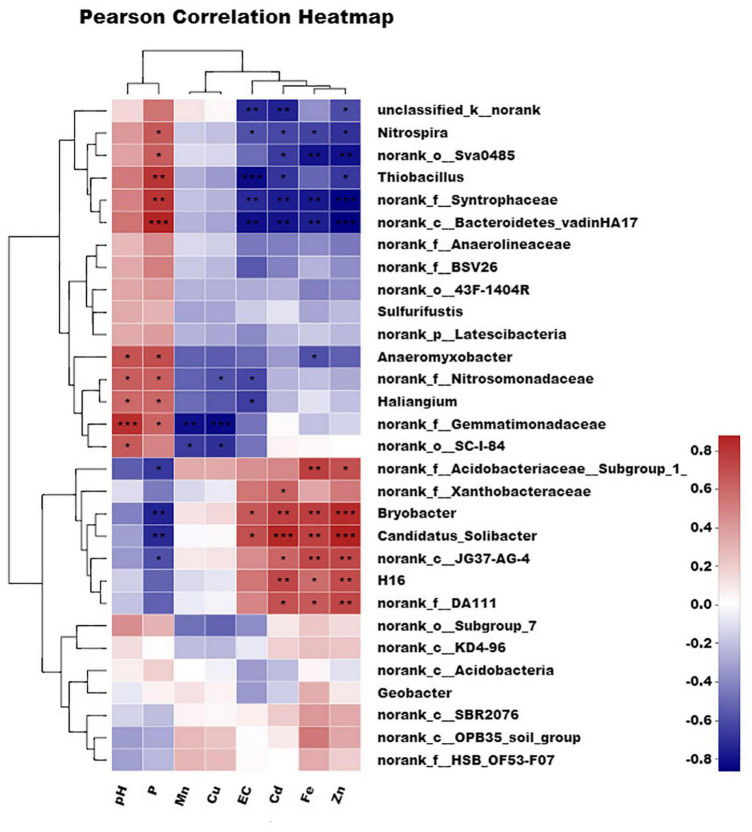
Correlation between different influencing factors and different microorganisms in genus level. Single asterisk (*P < 0.05), double asterisk (**P < 0.01), and three asterisk (***P ≤ 0.001) indicate statistically significant differences between values.

The detailed correlation between the organism at the genus level and environmental parameters is shown in Figure 6 and Supplementary Table 3. A genus-level heatmap split the eight parameters into three groups at the first level. One was composed of pH and phosphorous, another was composed of manganese and copper, and the last was composed of EC, cadmium, iron, and zinc. The effect of parameters such as pH and phosphorous on organisms was greatly different from that of EC, cadmium, iron, and zinc. It is corresponded to the results of the canonical correspondence analysis (CCA).

**FIGURE 7:**
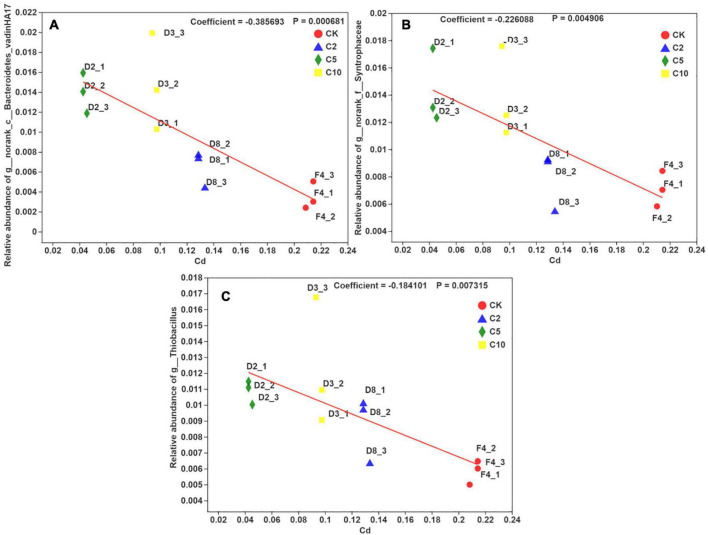
The multivariate association with linear models between the relative abundance of three microorganisms **(A–C)** in the genus level and available Cd concentration in soil with different amendment concentration.

*Bacterodietes*-*vadinHA17*, *Syntrophaceae*, and *Thiobacillus* were significantly positively correlated (*R* = 0.80, *P* ≤ 0.001) with phosphorous, while *Bryobacter* and *Candidatus-Solibacter* were significantly negatively correlated with phosphorous (*R* =–0.70, *P* ≤ 0.001). The opposite relationship was present between phosphorous and pH, and cadmium, iron, zinc, and EC. As a result, *Bacterodietes*-*vadinHA17* and *Syntrophaceae* were negatively correlated with cadmium, iron, zinc, and EC. However, *Bryobacter*, *Candidatus-Solibacter*, and *H16* were positively correlated with these four parameters. Usually, *Thiobacillus thiooxidans* can produce substantial amounts of H^+^ and then induce low pH. The treatment with high content FB7 induced the relatively low bioavailable iron, manganese, and zinc for paddy soil. The multivariate association with linear models for three typical microorganisms (*Bacterodietes*-*vadinHA17*, *Syntrophaceae*, and *Thiobacillus*) and the available cadmium concentration in soil is shown in Figure 7. The relationship coefficients in the linear model were –0.38, –0.22, and –0.18 for *Bacterodietes*-*vadinHA17*, *Syntrophaceae*, and *Thiobacillus*, respectively.

*Bacterodietes*-*vadinHA17* is a typical organic matter-degradation bacterium (Yang J. et al., 2018). Baldwin found the order *Bacteroidetes* environmental group vadinHA17 in environments undergoing complex carbon degradation, especially in samples with relatively high amounts of recalcitrant organic matter (Baldwin et al., 2015). Similarly, *Syntrophaceae* usually play an important part in the methanogenic hexadecane degradation (Lei et al., 2013). Gray et al. (2011) revealed a significant positive correlation between *Syntrophaceae*-affiliated clones and methane production from a petroleum hydrocarbon-degrading consortium. However, it has not been reported if the resulting high concentration P can increase the abundance of *Bacterodietes*-*vadinHA17* and *Syntrophaceae*. The present result may imply that adding a higher concentration of phosphorous to the soil can induce more organic matter to degrade into more methane. However, the detailed mechanism on the relationship needs further investigation.

*Thiobacillus thiooxidans* reduced soil pH and increased the uptake of iron, manganese, and zinc in paddy roots and shoots (Aria et al., 2010). This result may correspond to a lower pH in the C10 than the C5 treatment. It was reported that *Thiobacillus thiooxidans* can be subcultivated in the environment with a high concentration of cadmium, through the formation of a cadmium-complex (Sakamoto et al., 1989). Thus, the low bioavailable cadmium may be attributed to the high abundance of *Thiobacillus* in the soil.

Bacteria in the genus *Bryobacter* and *Candidatus-Solibacter* belong to the Acidobacteria phylum, which is correlated with H^+^ production in soil. These two bacteria can decrease with increasing nitrogen fertilizer due to lower soil pH after the application of more nitrogen fertilizer (Wang et al., 2017). However, in our experiment, the addition of a more alkaline substance such as CMP and lime caused higher pH (>7.2) long-term in the soil environment and then decreased these bacteria’s abundance. The growth of *Bryobacter* occurs at pH 4.5–7.2 (optimum growth at pH 5.5–6.5) (Kulichevskaya et al., 2010). Therefore, the presence of an appropriate growth pH may be the main effect on the abundance of Acidobacteria. However, *Bryobacter* and *Candidatus-Solibacter* showed a highly positive relationship with available metal ions such as cadmium, iron, and zinc. These bacteria can cause low pH and lots of fatty acid, a type of organic matter with few molecules (Kawai et al., 2012). Therefore, the higher abundance of *Bryobacter* and *Candidatus-Solibacter* induces higher bioavailable metal content.

The bacteria *norank-f-Gemmatimonadaceae* was significantly positively correlated to pH and negatively correlated with manganese and copper. It preferred the high pH environment and can reduce the bioavailability of manganese and copper. The bacteria *norank-f-Gemmatimonadaceae* has a polyphosphate-accumulating function (Takaichi et al., 2010). Therefore, low bioavailable manganese and copper might be attributed to the coprecipitation effect of the phosphate-metal ion (manganese, copper).

## Conclusion

Application of 0.5‰ CMP + lime amendment could mitigate soil acidity, reduce bioavailable cadmium, and decrease cadmium content in brown rice to the national food safety standard. Enough calcium, magnesium, manganese, and zinc were supplied for rice, although this amendment greatly decreased the iron, copper, and cadmium bioavailable content in soil. There will be a competition between those cations and cadmium ions, but the application of a small amount of amendment can promote the uptake of heavy metals in rice. The amendment can remediate the ecology of the soil and increase biodiversity. Specifically, the abundance of *Bacterodietes*-*vadinHA17*, *Syntrophaceae*, and *Thiobacillus* was significantly positively correlated with phosphorous, while *Bryobacter* and *Candidatus-Solibacter* were significantly negatively correlated with phosphorous. These microorganisms may potentially affect metal bioavailability and then decrease the cadmium uptake by the rice. Therefore, the CMP + lime amendment is recommended as a highly effective amendment for remediating cadmium-polluted acidic paddy soils in south China.

## Data Availability Statement

The original contributions presented in the study are publicly available. This data can be found here: National Center for Biotechnology Information (NCBI) BioProject database under accession number PRJNA761207.

## Ethics Statement

All procedures performed in studies involving human participants were in accordance with the ethical standards of the institutional and/or national research committee.

## Author Contributions

XK and KS conducted the whole experiment, did the analysis, and wrote the initial draft. HS and AC (ranked in order of their actual contribution) provided necessary facilities for the successful completion of this work. LP provided partial research grants for this study, proofread the manuscript, and also supervised the work. All authors contributed to the article and approved the submitted version.

## Conflict of Interest

The authors declare that the research was conducted in the absence of any commercial or financial relationships that could be construed as a potential conflict of interest.

## Publisher’s Note

All claims expressed in this article are solely those of the authors and do not necessarily represent those of their affiliated organizations, or those of the publisher, the editors and the reviewers. Any product that may be evaluated in this article, or claim that may be made by its manufacturer, is not guaranteed or endorsed by the publisher.

## References

[B1] Aria M. M. Lakzian A. Haghnia G. H. Berenji A. R. Besharati H. Fotovat A. (2010). Effect of *Thiobacillus*, sulfur, and vermicompost on the water-soluble phosphorus of hard rock phosphate. *Bioresour. Technol.* 101 551–554. 10.1016/j.biortech.2009.07.093 19736005

[B2] Baldwin S. A. Khoshnoodi M. Rezadehbashi M. Taupp M. Hallam S. Mattes A. (2015). The microbial community of a passive biochemical reactor treating arsenic, zinc, and sulfate-rich seepage. *Front. Bioeng. Biotechnol.* 3:27. 10.3389/fbioe.2015.00027 25798439 PMC4351619

[B3] Bouray M. Moir J. L. Condron L. M. Lehto N. J. (2021). Lime-induced ph elevation influences phosphorus biochemical processes and dynamics in the rhizosphere of *Lupinus polyphyllus* and *Lupinus angustifolius*. *J. Soil Sci. Plant Nutr.* 21 1978–1992. 10.1007/s42729-021-00495-z

[B4] Cai Y. Xu W. Wang M. Chen W. Li X. Li Y. (2019). Mechanisms and uncertainties of Zn supply on regulating rice Cd uptake. *Environ. Pollut.* 253 959–965. 10.1016/j.envpol.2019.07.077 31351304

[B5] Gong L. Wang J. Abbas T. Zhang Q. Di H. (2021). Immobilization of exchangeable Cd in soil using mixed amendment and its effect on soil microbial communities under Paddy upland rotation system. *Chemosphere* 262:127828. 10.1016/j.chemosphere.2020.127828 32763579

[B6] Gray N. D. Sherry A. Grant R. J. Rowan A. K. Hubert C. R. J. Callbeck C. M. (2011). The quantitative significance of Syntrophaceae and syntrophic partnerships in methanogenic degradation of crude oil alkanes. *Environ. Microbiol.* 13 2957–2975. 10.1111/j.1462-2920.2011.02570.x 21914097 PMC3258425

[B7] Guha T. Barman S. Mukherjee A. Kundu R. (2020). Nano-scale zero valent iron modulates Fe/Cd transporters and immobilizes soil Cd for production of Cd free rice. *Chemosphere* 260:127533. 10.1016/j.chemosphere.2020.127533 32679374

[B8] Han C. Lalley J. Iyanna N. Nadagouda M. N. (2017). Removal of phosphate using calcium and magnesium-modified iron-based adsorbents. *Mater. Chem. Phys.* 198 115–124. 10.1016/j.matchemphys.2017.05.038

[B9] Heijs S. K. Aloisi G. Bouloubassi I. Pancost R. D. Pierre C. Damsté J. S. S. (2006). Microbial community structure in three deep-sea carbonate crusts. *Microb. Ecol.* 52 451–462. 16909345 10.1007/s00248-006-9099-8

[B10] Karaca A. Naseby D. C. Lynch J. M. (2002). Effect of cadmium contamination with sewage sludge and phosphate fertiliser amendments on soil enzyme activities, microbial structure and available cadmium. *Biol. Fertil. Soils* 35 428–434. 10.1007/s00374-002-0490-4

[B11] Kawai K. Takato S. Sasaki T Kajiwara K. (2012). Complex formation, thermal properties, and *in-vitro* digestibility of gelatinized potato starch–fatty acid mixtures. *Food Hydrocoll.* 27 228–234. 10.1016/j.foodhyd.2011.07.003

[B12] Kulichevskaya I. S. Suzina N. E. Liesack W. Dedysh S. N. (2010). *Bryobacter aggregatus* gen. nov., sp. nov., a peat-inhabiting, aerobic chemo-organotroph from subdivision 3 of the Acidobacteria. *Int. J. Syst. Evol. Microbiol.* 60 301–306. 10.1099/ijs.0.013250-0 19651730

[B13] Lei C. Chen D. Qiang L. Qiao H. Li-rong D. Hui Z. (2013). DNA-SIP reveals that syntrophaceae play an important role in methanogenic hexadecane degradation. *PLoS One* 8:e66784. 10.1371/journal.pone.0066784 23840866 PMC3698093

[B14] Li H. Abbas T. Cai M. Zhang Q. Tahir M. (2021). Cd bioavailability and nitrogen cycling microbes interaction affected by mixed amendments under Paddy-Pak Choi continued planting. *Environ. Pollut.* 275:116542. 10.1016/j.envpol.2021.116542 33582635

[B15] Li H. Liu Y. Zhou Y. Zhang J. Mao Q. Yang Y. (2018). Effects of red mud based passivator on the transformation of Cd fraction in acidic Cd-polluted paddy soil and Cd absorption in rice. *Sci. Total. Environ.* 640–641 736–745. 10.1016/j.scitotenv.2018.05.327 29879662

[B16] Li Y. Liang X. Huang Q. Xu Y. Yang F. (2019). Inhibition of Cd accumulation in grains of wheat and rice under rotation mode using composite silicate amendment. *RSC Adv.* 9 35539–35548. 10.1039/c9ra07137g PMC9074415 35528060

[B17] Lin C. Lu W. Wu Y. (2005). Agricultural soils irrigated with acidic mine water: acidity, heavy metals, and crop contamination. *Soil Res.* 43 819–826. 10.1071/sr04148

[B18] Liu L. Liu Y. Peng Z. Song G. Rong H. Wang Z. (2017). Development of bacterial communities in biological soil crusts along a revegetation chronosequence in the Tengger Desert, northwest China. *Biogeosciences* 14 1–25.

[B19] Liu Y. Zhang C. Zhao Y. Sun S. Liu Z. (2018). Effects of growing seasons and genotypes on the accumulation of cadmium and mineral nutrients in rice grown in cadmium contaminated soil. *Sci. Total. Environ.* 579 1282–1288. 10.1016/j.scitotenv.2016.11.115 27908623

[B20] Luo W. Yang S. Khan M. A. Ma J. Liu D. (2020). Mitigation of Cd accumulation in rice with water management and calcium-magnesium phosphate fertilizer in field environment. *Environ. Geochem. Health* 42 3877–3886. 32617850 10.1007/s10653-020-00648-6

[B21] Mao P. Zhuang P. Li F. McBride M. B. Ren W. Li Y. (2019). Phosphate addition diminishes the efficacy of wollastonite in decreasing Cd uptake by rice (*Oryza sativa* L.) in paddy soil. *Sci. Total Environ.* 687 441–450. 10.1016/j.scitotenv.2019.05.471 31212152

[B22] Mcbride M. Sauve S. Hendershot W. (2010). Solubility control of Cu, Zn, Cd and Pb in contaminated soils. *Eur. J. Soil Sci.* 48 337–346. 10.1111/j.1365-2389.1997.tb00554.x

[B23] Peng L. Xu Y. Zhou F. Sun B. R. Tie B. Q. (2016). Enhanced removal of Cd(II) by poly(acrylamide-co-sodium acrylate) water-retaining agent incorporated nano hydrous manganese oxide. *Mater. Des.* 96 195–202.

[B24] Rehman M. Rizwan M. Rauf A. Ayub M. A. Ali S. Qayyum M. F. (2019). Split application of silicon in cadmium (Cd) spiked alkaline soil plays a vital role in decreasing Cd accumulation in rice (*Oryza sativa* L.) grains. *Chemosphere* 226 454–462. 30951940 10.1016/j.chemosphere.2019.03.182

[B25] Rosa D. J. Ambrosini V. G. Kokkoris V. Brunetto G. Hart M. Ricachenevsky F. (2020). Lime protection for young vines exposed to copper toxicity. *Water Air Soil Pollut.* 231 1–10.

[B26] Saengwilai P. Meeinkuirt W. (2021). Cadmium (Cd) and zinc (Zn) accumulation by Thai rice varieties and health risk assessment in a Cd–Zn co-contaminated paddy field: effect of soil amendments. *Environ. Geochem. Health* 43 3659–3674. 10.1007/s10653-021-00858-6 33630197

[B27] Sakamoto K. Yagasaki M Kirimura K Usami S. (1989). Resistance acquisition of *Thiobacillus thiooxidans* upon cadmium and zinc ion addition and formation of cadmium ion-binding and zinc ion-binding proteins exhibiting metallothionein-like properties. *J. Ferment. Bioeng.* 67 266–273. 10.1016/0922-338X(89)90230-4

[B28] Shi R. Y. Ni N. Nkoh J. N. Dong Y. Zhao W. R. Pan X. Y. (2020). Biochar retards Al toxicity to maize (*Zea mays* L.) during soil acidification: the effects and mechanisms. *Sci. Total Environ.* 719:137448. 10.1016/j.scitotenv.2020.137448 32112949

[B29] Singh P. Pokharia C. Shah K. (2021). Exogenous peroxidase mitigates cadmium toxicity, enhances rhizobial population and lowers root knot formation in rice seedlings. *Rice Sci.* 28 166–177. 10.1016/j.rsci.2021.01.006

[B30] Siqi T. Shuang L. Kun Q. Fanhong W. Yuxiu Z. Chai T. (2019). Co-expression of multiple heavy metal transporters changes the translocation, accumulation, and potential oxidative stress of Cd and Zn in rice (*Oryza sativa*). *J. Hazard. Mater.* 380:120853. 10.1016/j.jhazmat.2019.120853 31279944

[B31] Song H. Peng L. Li Z. Deng X. Shao J. Gu J. D. (2019). Metal distribution and biological diversity of crusts in paddy fields polluted with different levels of cadmium. *Ecotoxicol. Environ. Saf.* 184 1–8. 10.1016/j.ecoenv.2019.109620 31493587

[B32] Stroud J. Low A. Collins R. N. Manefield M. (2014). Metal(loid) bioaccessibility dictates microbial community composition in acid sulfate soil horizons and sulfidic drain sediments. *Environ. Sci. Technol.* 48 8514–8521. 10.1021/es501495s 25000450

[B33] Sun G. X. Zhang L. Chen P. Yao B. M. (2020). Silicon fertilizers mitigate rice cadmium and arsenic uptake in a 4-year field trial. *J. Soils Sediments* 21 163–171.

[B34] Takaichi S. Maoka T. Takasaki K. Hanada S. (2010). Carotenoids of *Gemmatimonas aurantiaca* (Gemmatimonadetes): identification of a novel carotenoid, deoxyoscillol 2-rhamnoside, and proposed biosynthetic pathway of oscillol 2,2-dirhamnoside. *Microbiology* 156 757–763. 10.1099/mic.0.034249-0 19959572

[B35] Wang C. Huang Y. Zhang C. Zhang Y. Liu Y. Liu Z. (2021). Inhibition effects of long-term calcium-magnesia phosphate fertilizer application on Cd uptake in rice: regulation of the iron-nitrogen coupling cycle driven by the soil microbial community. *J. Hazard. Mater.* 416:125916. 10.1016/j.jhazmat.2021.125916 34492849

[B36] Wang D. Z. Jiang X. Rao W. He J. Z. (2009). Kinetics of soil cadmium desorption under simulated acid rain. *Ecol. Complex.* 6 432–437. 10.1016/j.ecocom.2009.03.010

[B37] Wang L. Ashraf U. Chang C. Abrar M. Cheng X. (2020). Effects of silicon and phosphatic fertilization on rice yield and soil fertility. *J. Soil Sci. Plant Nutr.* 20 557–565.

[B38] Wang R. Xiao Y. Lv F. Hu L. Wei L. Yuan Z. (2017). Bacterial community structure and functional potential of rhizosphere soils as influenced by nitrogen addition and bacterial wilt disease under continuous sesame cropping. *Appl. Soil Ecol.* 125 117–127.

[B39] Xie Y. Wang J. Wu Y. Ren C. Song C. Yang J. (2016). Using in situ bacterial communities to monitor contaminants in river sediments. *Environ. Pollut.* 212 348–357. 26866572 10.1016/j.envpol.2016.01.031

[B40] Xu Y. Feng J. Li H. (2021). How intercropping and mixed systems reduce cadmium concentration in rice grains and improve grain yields. *J. Hazard. Mater.* 402:123762. 10.1016/j.jhazmat.2020.123762 33254775

[B41] Yang Y. Chen J. Huang Q. Tang S. Wang J. Hu P. (2018). Can liming reduce cadmium (Cd) accumulation in rice (*Oryza sativa*) in slightly acidic soils? A contradictory dynamic equilibrium between Cd uptake capacity of roots and Cd immobilisation in soils. *Chemosphere* 193 547–556. 10.1016/j.chemosphere.2017.11.061 29169130

[B42] Yang J. Pengfei L. Ralf C. (2018). Response of fermenting bacterial and methanogenic archaeal communities in paddy soil to progressing rice straw degradation. *Soil Biol. Biochem.* 124 70–80.

[B43] Yang J. Wang C. Dai H. (2008). *Soil Agrochemical Analysis and Environmental Monitoring.* Beijing: China Earth Press.

[B44] Yin H. Yang C. Yang P Kaksonen A. H. Douglas G. B. (2021). Contrasting effects and mode of dredging and *in situ* adsorbent amendment for the control of sediment internal phosphorus loading in eutrophic lakes. *Water Res.* 189 116644–116654. 10.1016/j.watres.2020.116644 33221586

[B45] Zhang Y. Wang X. Ji X. Liu Y. Lin Z. Lin Z. (2019). Effect of a novel Ca-Si composite mineral on Cd bioavailability, transport and accumulation in paddy soil-rice system. *J. Environ. Manage.* 233 802–811. 10.1016/j.jenvman.2018.10.006 30446285

[B46] Zhao F. J. Ma Y. Zhu Y. G. Tang Z. Mcgrath S. P. (2015). Soil contamination in China: current status and mitigation strategies. *Environ. Sci. Technol.* 49 750–759. 10.1021/es5047099 25514502

[B47] Zhen Z. Wang S. Luo S. Ren L. Liang Y. Yang R. (2019). Significant impacts of both total amount and availability of heavy metals on the functions and assembly of soil microbial communities in different land use patterns. *Front. Microbiol.* 10:2293. 10.3389/fmicb.2019.02293 31636621 PMC6788306

[B48] Zhu J. Zhang J. Li Q. Han T. Xie J. Hu Y. (2013). Phylogenetic analysis of bacterial community composition in sediment contaminated with multiple heavy metals from the Xiangjiang River in China. *Mar. Pollut. Bull.* 70 134–139. 23507235 10.1016/j.marpolbul.2013.02.023

