# Supplementary figures and images for: Lime-Phosphorus Fertilizer Efficiently Reduces the Cd Content of Rice: Physicochemical Property and Biological Community Structure in Cd-Polluted Paddy Soil
Source: Front Microbiol. 2021 Nov 19;12:749946. doi: 10.3389/fmicb.2021.749946 (PMC8638080; doi:10.3389/fmicb.2021.749946)

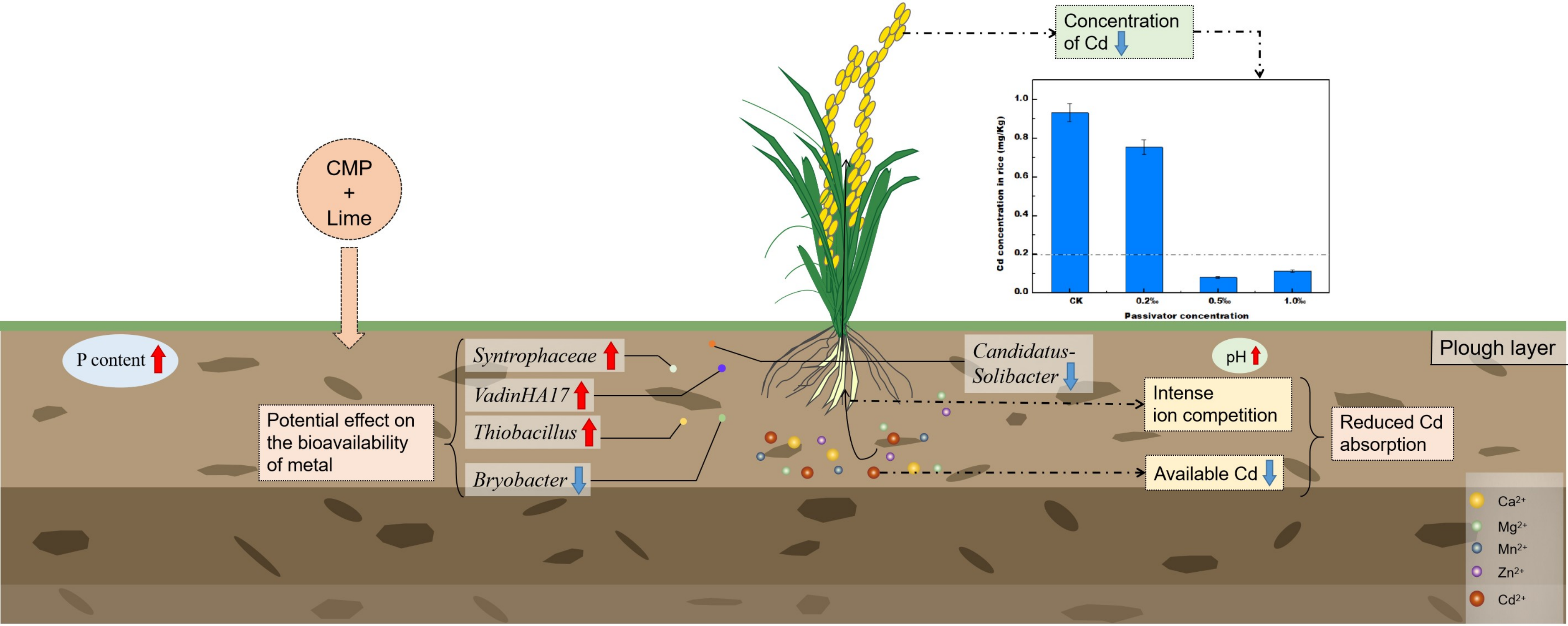

Supplement: Supplementary file 1 [file Presentation_1.PDF]
