# Supplementary material for: Lime-Phosphorus Fertilizer Efficiently Reduces the Cd Content of Rice: Physicochemical Property and Biological Community Structure in Cd-Polluted Paddy Soil
Source: Front Microbiol. 2021 Nov 19;12:749946. doi: 10.3389/fmicb.2021.749946 (PMC8638080; doi:10.3389/fmicb.2021.749946)
Supplement: Supplementary file 2 [file Table_1.DOC]

1. 0.5‰ concentration of amendment effectively reduced 88.2% cadmium in rice.
2. The abundance of *Bacterodietes-vadinHA17*, *Syntrophaceae* and *Thiobacillus* was significant positive correlation with P.
3. The application of 0.2‰ amount of amendment can promote the absorption of heavy metals in rice.
