# Supplementary material for: Lime-Phosphorus Fertilizer Efficiently Reduces the Cd Content of Rice: Physicochemical Property and Biological Community Structure in Cd-Polluted Paddy Soil
Source: Front Microbiol. 2021 Nov 19;12:749946. doi: 10.3389/fmicb.2021.749946 (PMC8638080; doi:10.3389/fmicb.2021.749946)
Supplement: Supplementary file 3 [file Table_2.DOC]

**Supported Information**

**Phosphorus Fertilizer Efficiently Reduces the Cd Content of Rice: Physicochemical Proper****ty and Biological** **Community Structure in Cd-polluted Paddy Soil**

**Xiaolin Kuang^1,2^ Kangying Si^1,2^, Huijuan Song^1,2^,** **Liang Peng^1,2*^, Anwei Chen^1,2^**

^1^Department of Environmental Science & Engineering, Hunan Agricultural University, Changsha 410128, P. R. China

^2^Hunan Engineering and Technology Research Center for Irrigation Water Purification, Changsha 410128, P. R. China

* Corresponding author at: College of Resource and Environment, Hunan Agricultural University, Changsha, 410128, China, Tel: +86 0731 84617803

E-mail: pengliang2004@126.com; pengliang@hunau.edu.cn (L.Peng)

**1 Bacterial richness and diversity**

The PC samples from three locations at every contamination level were mixed together and thoroughly homogenized. For each, its total microbial DNA was extracted using a Soil Master DNA Extraction kit (Epicentre Biotechnologies, Madison, WI, USA), according to the manufacturer’s instructions. The universal primers 515F (GTGCCAGCMGCCGCGG) and 907R (CCGTCAATTCMTTTRAGTTT) were used to amplify the V4-V5 region of the 16S ribosomal RNA (rRNA) gene (i.e., 515–907).

The oligonucleotide sequence barcode was fused to the forward primer. The PCR reaction mixture (20 μL) contained 4 μL of 5× FastPfu reaction buffer (TransGen Biotech, Beijing, China); 2 μL of a dNTP mixture (2.5 mM); 0.4 μL of each primer (5 μmol/L); 0.4 μL of FastPfu polymerase; 10 ng of the template DNA, with H_2_O added to make up the volume. For the PCR thermal cycling, an applied microbiology biotechnology scheme was set as follows: initial denaturation at 95°C for 5 min, 25 cycles of denaturation at 95°C for 30 s, annealing at 55°C for 30 s, and an extension at 72°C for 30 s, followed by a final extension period of 5 min at 72°C. This PCR amplification was performed on an ABI GeneAmp PCR System 9700 (Applied Biosystems, Foster City, CA, USA) and its ensuing products examined on 2% (w/v) agarose gel and further purified with an Maxiprep DNA Gel Extraction Kit (Axygen Biosciences, Union City, CA, USA). Purified amplicons were quantified using QuantiFluor™-ST (Promega, Fitchburg, WI, USA) with their paired-end sequencing carried out on an Illumina MiSeq platform at Majorbio Bio-Pharm Technology Co., Ltd. (Shanghai, China), according to standard protocols.

These tags were clustered to operational taxonomic units (OTUs) using a threshold of 97% sequence similarity. Taxonomic ranks were assigned to OTU representative sequences by using the naïve Bayesian Classifier (v.2.2) of the Ribosomal Database Project (RDP). Finally, the diversity was determined, and the different bacterial species were screened based on their OTUs and taxonomic rankings.

**2 Bioinformatics and statistical analysis**

Raw pyrosequencing data were de-multiplexed and quality-filtered, by using Trimmomatic tool in the way Lohse et al. (2012) described. Overlapping reads were then merged into single long reads with the FLASH software tool (Mago and Salzberg, 2011). Qualified sequences were then clustered into OTUs at a 97% similarity cutoff using Usearch v7.1 (http://qiime.org/). The phylogenetic affiliation of each 16S rDNA sequence was analyzed with the RDP Classifier v2.2 (http://sourceforge.net/projects/rdp-classifier/), using a confidence threshold of 0.7 and the reference database Silva (Release 115, http://www.arb-silva.de). Venn diagrams and heatmap figures were produced using package 'gplots' in the R (v3.1.1) software (http://www.Rproject.org/). A Canonical correspondence analysis (CCA) was performed using Canoco v.5.0 software (Microcomputer Power, Ithaca, NY, USA).

**Reference**

1. Lohse, M., Bolger, A.M., Nagel, A., Fernie, A.R., Lunn, J.E., Stitt, M., Usadel, B., 2012. RobiNA: a user-friendly, integrated software solution for RNA-Seq-based transcriptomics. Nucleic Acids Research 40, W622.
2. Mago, T., Salzberg, S.L., 2011. FLASH: fast length adjustment of short reads to improve genome assemblies. Bioinformatics 27, 2957-2963.

**
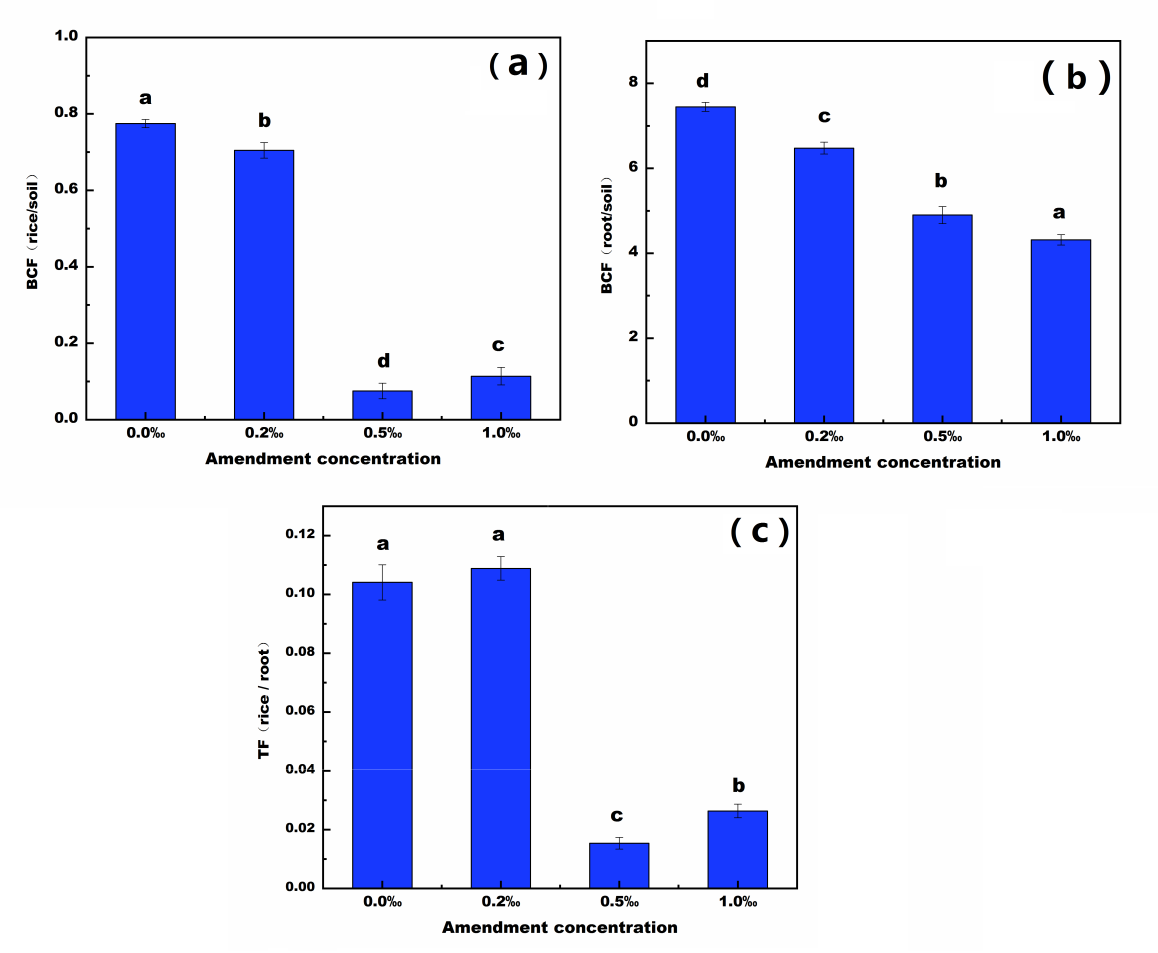
**

**Fig.S1** Effects of different treatments on translocation factor and bioconcentration factors of rice


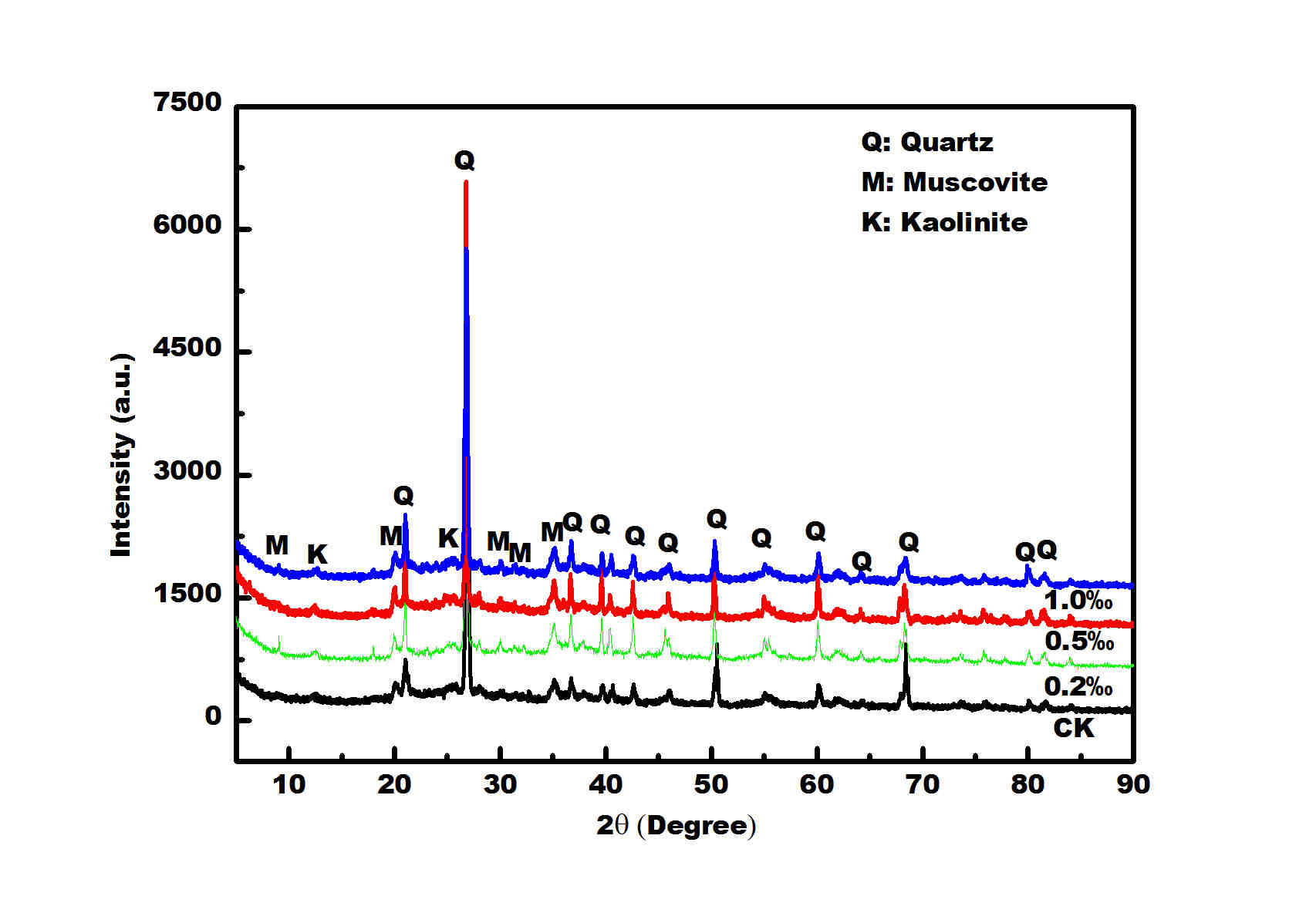


**Fig.S2** the XRD of the soil samples processed with 0, 0.2, 0.5, 1.0‰ passivating agent.


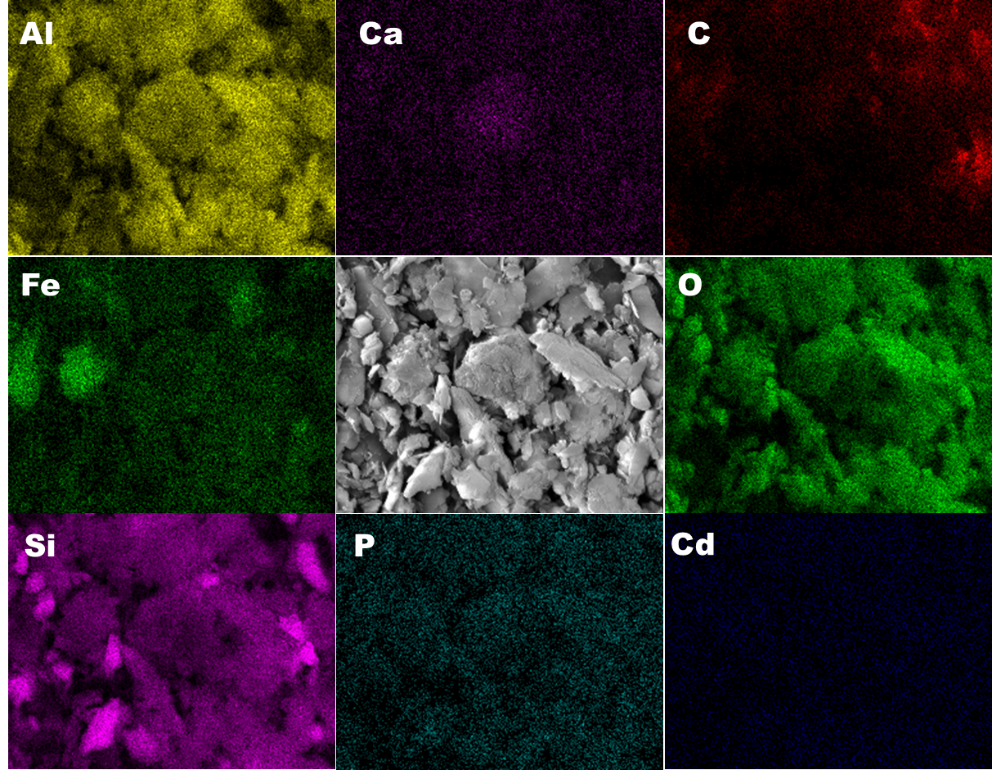


**Fig.S3** the element mapping of the soil from samples without passivating agent addition.


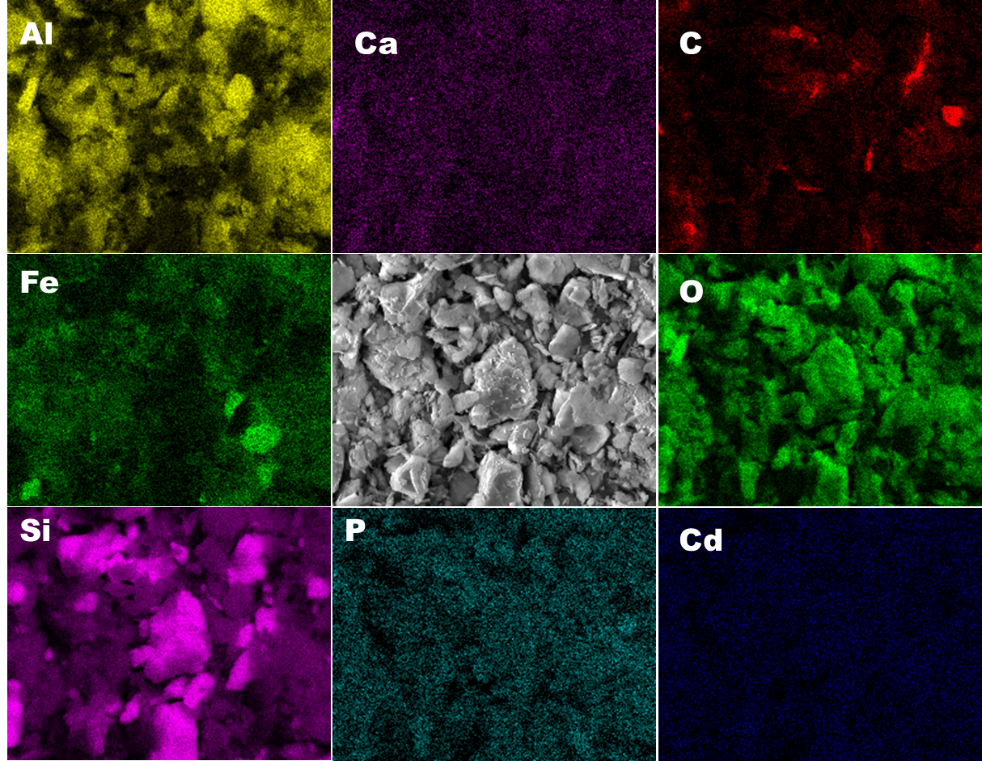


**Fig.S4** the element mapping of the soil from samples proceed with 1.0‰ passivating agent.

**Table S1 Rice effective tiller, biomass, yield, seed setting rate and 1000-grain weight under different treatments**

| Treatment | Effective tiller number per plant | Biomass（kg/hm^2^） | Yield（kg/hm^2^） | Seed setting rate（‰） | 1000-grain weight（g） |
| --- | --- | --- | --- | --- | --- |
| CK | 12.36±0.12 c | 11996.73±72.21 d | 7596.73±62.37 d | 82.34±0.63 b | 22.31±0.89 a |
| 0.2‰ | 12.76±0.23 b | 12287.62±66.98 c | 7887.62±56.31 c | 83.98±0.14 a | 22.82±0.66 a |
| 0.5‰ | 13.16±0.14 a | 12980.89±84.77 b | 7950.89±74.44 bc | 84.02±0.57 a | 22.83±1.14 a |
| 1‰ | 13.36±0.05 a | 13121.45±53.87 a | 8021.45±63.19 ab | 84.63±0.44 a | 23.34±0.98 a |

**Table S2** The content of calcium and available phosphorus in the soil after  application of amendments

| Treatment | Ca（g/kg） | Olsen-P（mg/kg） |
| --- | --- | --- |
| CK | 1.56±0.02 d | 29.78±0.92 d |
| 0.2‰ | 1.77±0.08 c | 30.65±0.04 c |
| 0.5‰ | 1.95±0.06 b | 32.19±0.55 b |
| 1‰ | 2.37±0.05 a | 35.36±0.72 a |

**Table S3** Available metal concentration in the soil

| Treatment | Fe/g kg^-1^ | Mn/mg kg^-1^ | Zn/mg kg^-1^ | Cu/mg kg^-1^ |
| --- | --- | --- | --- | --- |
| CK | 2.58±0.35 b | 241.50±11.6 b | 76.29±3.54 a | 2.50±0.19 a |
| 0.2‰ | 3.25±0.12 a | 269.21±7.98 a | 69.29±4.18 b | 2.67±0.14 a |
| 0.5‰ | 1.19±0.08 c | 280.31±6.74 a | 15.63±1.28 d | 2.81±0.15 a |
| 1.0‰ | 1.50±0.11 c | 203.75±9.41 c | 29.54±2.69 c | 2.08±0.22 b |

Note: CK is blank control

**Table S4 The correlation coeffiecne of bioorganism in phylum level with environmental factor**

| **Species** | **pH** | **P** | **Mn** | **Cu** | **EC** | **Cd** | **Fe** | **Zn** |
| --- | --- | --- | --- | --- | --- | --- | --- | --- |
| Nitrospira | 0.41 | 0.66 | -0.17 | -0.21 | -0.59 | -0.62 | -0.63 | -0.69 |
| norank_f__Anaerolineaceae | 0.29 | 0.46 | -0.12 | -0.15 | -0.45 | -0.43 | -0.38 | -0.45 |
| norank_c__Acidobacteria | 0.07 | 0.19 | 0.01 | -0.04 | -0.33 | -0.22 | 0.05 | -0.09 |
| norank_c__SBR2076 | -0.14 | -0.21 | 0.06 | 0.04 | 0.07 | 0.22 | 0.42 | 0.35 |
| norank_f__Acidobacteriaceae__Subgroup | -0.54 | -0.67 | 0.34 | 0.35 | 0.44 | 0.48 | 0.76 | 0.70 |
| H16 | -0.16 | -0.53 | -0.12 | -0.08 | 0.55 | 0.73 | 0.58 | 0.71 |
| norank_o__Subgroup_7 | 0.45 | 0.31 | -0.48 | -0.53 | -0.38 | 0.10 | 0.23 | 0.15 |
| norank_f__BSV26 | 0.34 | 0.51 | -0.18 | -0.23 | -0.56 | -0.42 | -0.25 | -0.38 |
| norank_f__Gemmatimonadaceae | 0.82 | 0.62 | -0.81 | -0.83 | -0.46 | 0.03 | -0.20 | -0.14 |
| ulfurifustis | 0.34 | 0.31 | -0.30 | -0.29 | -0.17 | -0.09 | -0.29 | -0.22 |
| norank_o__Sva0485 | 0.37 | 0.64 | -0.12 | -0.13 | -0.49 | -0.66 | -0.79 | -0.80 |
| norank_f__Nitrosomonadaceae | 0.64 | 0.64 | -0.53 | -0.58 | -0.62 | -0.24 | -0.21 | -0.28 |
| Geobacter | -0.07 | 0.06 | 0.13 | 0.06 | -0.34 | -0.16 | 0.33 | 0.10 |
| norank_o__SC-I-84 | 0.66 | 0.49 | -0.66 | -0.70 | -0.47 | 0.05 | 0.04 | 0.01 |
| Bryobacter | -0.42 | -0.74 | 0.12 | 0.16 | 0.66 | 0.76 | 0.77 | 0.84 |
| Candidatus_Solibacter | -0.32 | -0.72 | -0.01 | 0.04 | 0.70 | 0.86 | 0.75 | 0.88 |
| norank_c__KD4-96 | 0.15 | 0.02 | -0.21 | -0.23 | -0.07 | 0.19 | 0.26 | 0.24 |
| norank_f__Xanthobacteraceae | -0.11 | -0.44 | -0.13 | -0.07 | 0.55 | 0.63 | 0.36 | 0.53 |
| norank_p__Latescibacteria | 0.34 | 0.39 | -0.25 | -0.29 | -0.39 | -0.22 | -0.17 | -0.23 |
| unclassified_k__norank | 0.16 | 0.55 | 0.12 | 0.04 | -0.71 | -0.74 | -0.36 | -0.60 |
| norank_c__OPB35_soil_group | -0.33 | -0.28 | 0.28 | 0.24 | 0.00 | 0.09 | 0.53 | 0.36 |
| Anaeromyxobacter | 0.68 | 0.70 | -0.53 | -0.54 | -0.50 | -0.34 | -0.59 | -0.54 |
| norank_f__Syntrophaceae | 0.50 | 0.81 | -0.20 | -0.25 | -0.72 | -0.76 | -0.78 | -0.85 |
| norank_c__Bacteroidetes_vadinHA17 | 0.56 | 0.87 | -0.24 | -0.30 | -0.81 | -0.79 | -0.76 | -0.86 |
| norank_o__43F-1404R | 0.36 | 0.41 | -0.25 | -0.25 | -0.27 | -0.25 | -0.42 | -0.38 |
| Thiobacillus | 0.53 | 0.80 | -0.26 | -0.34 | -0.83 | -0.68 | -0.51 | -0.67 |
| Haliangium | 0.60 | 0.61 | -0.50 | -0.56 | -0.65 | -0.23 | -0.09 | -0.21 |
| norank_f__DA111 | -0.20 | -0.53 | -0.06 | -0.03 | 0.48 | 0.69 | 0.66 | 0.73 |
| norank_f__HSB_OF53-F07 | -0.31 | -0.23 | 0.30 | 0.27 | 0.03 | 0.01 | 0.34 | 0.21 |
| norank_c__JG37-AG-4 | -0.34 | -0.59 | 0.10 | 0.12 | 0.46 | 0.61 | 0.72 | 0.74 |
